# Utilizing SSR-based core collection development to improve conservation and utilization of *Corylus* L. genetic resources

**DOI:** 10.1371/journal.pone.0312116

**Published:** 2024-10-29

**Authors:** Weicong Yang, Boning Yang, Liyuan Lu, Xuemei Zhang, Jun Sun, Liwei Wang, Zeyang Zheng, Dejun Liang, Kehan Wang, Xinyu Yan, Chenchen Yang, Zhenpan Liu

**Affiliations:** 1 Liaoning Institute of Economic Forestry, Dalian, Liaoning Province, China; 2 Forestry College of Southwest Forestry University, Kunming, Yunnan Province, China; 3 Linghai Forestry & Grassland Protection Centre, Jinzhou, Liaoning Province, China; Government College University Faisalabad, PAKISTAN

## Abstract

Hazelnuts are traditional woody oilseed plants. *Corylus* L. resources are rich in variety and widely distributed in China. However, the identification of germplasm varieties and the selection of superior varieties remain quite limited. This study aimed to analyze the genetic diversity of 331 *Corylus* L. germplasms using 16 simple sequence repeat (SSR) markers. Based on this, 11 pairs of core primers were selected, a fingerprint database of germplasm resources was constructed, and a primary core collection was screened. The results indicated that these tested *Corylus* L. germplasms exhibited a high level of genetic diversity, with an average number of alleles (*Na*) per locus of 14.5 and a polymorphic information content of 0.777. The phylogenetic relationships among various hazelnut cultivars were characterized by complexity, and they were delineated into four distinct groups facilitated by genetic distance analyses. An SSR fingerprint database for 331 *Corylus* L. germplasms was successfully constructed using the 11 obtained core SSR markers to increase the discrimination efficiency. Ultimately, 127 primary core accessions of *Corylus* L. were selected. The retention rate for the observed *Na* and MAF (the minor allele frequency) in the primary core germplasm constructed based on a sampling proportion of 38.36% was 100% and 94.7%, respectively. Shannon’s information index (*I*) was highly consistent between the core and original germplasms, indicating that the core germplasm could fully represent the genetic diversity of the original germplasm. Additionally, the principal coordinate analysis of the selected primary core germplasm was essentially consistent with that of the entire original germplasm, further supporting the broad representativeness of the core germplasm. This study provided a basis for precisely identifying and efficiently utilizing *Corylus* L. accession.

## Introduction

Hazel (*Corylus* spp.), a perennial deciduous shrub or dwarf tree belonging to the Corylaceae family, is a nut-producing species with significant cultivation value. Its high-quality production is crucial in forest land utilization and regional development. Traditionally esteemed for their medicinal and dietary benefits, filbert kernels can be consumed fresh, roasted, or pressed for oil. They are also used as a raw material for various food products. They hold extensive application potential and high utility value due to their versatility as fruit, oil, and grain.

The genus *Corylus* is endowed with a rich genetic resource base and a diverse array of cultivars, encompassing 25 species worldwide, with most germplasm resources concentrated in the northern temperate zone [1]. The diverse climatic regions in China facilitate a concentrated distribution of approximately 10 hazelnut species. The primary species cultivated on a large scale are the Ping’ou hybrid hazelnut (*C*. *heterophylla* Fisch. × *C*. *avellana* L.) and the European hazelnut (*C*. *avellana* L.), which are predominantly situated in the northeastern and northern regions north of the Qinling–Huaihe Line, within the latitudinal range of 32°to 43′N [2]. The cultivation performance of European hazelnuts is particularly significant when investigating the environmental and climatic adaptability of hazelnut species. Despite the ability of European hazelnut to grow normally in the northern subtropical and mid-subtropical northern border regions, its fruiting traits are subpar, not meeting the economic cultivation expectations, thereby limiting its promotion in regions south of the Yangtze River Basin in China [3]. However, the European hazelnut still demonstrates certain adaptability and developmental potential in localized areas with microclimatic conditions, providing room for cultivation and variety creation in these regions. Since the 1980s, China has employed superior seedlings of European hazelnut varieties introduced from Europe as the paternal parent and selected outstanding individual plants of the native *C*. *heterophylla* as the maternal parent for hybrid breeding [4]. This breeding approach helped develop several hybrid varieties, including Dawei, Yuzhui, and the Liao Zhen 1–9 series, which are hybrids of *C*. *heterophylla* Fisch. × *C*. *avellana* L. These hybrids have become the predominant hazelnut cultivars in China. They are distinguished by their large nut size, high yield, strong adaptability, resistance to poor soil conditions, and effective soil and water conservation properties, thereby aligning well with the market demands. A national hazelnut germplasm repository was established in Jinzhou, Liaoning Province, to systematically preserve and effectively utilize these diverse germplasm resources, which currently houses 552 hazelnut germplasm resources. This includes 116 Ping’ou hybrid hazelnut varieties developed through seeding selection and cross-breeding. Most of these preserved hazelnut accessions possess excellent qualities, adaptability, and high productivity, thereby ensuring the sustainable use of genetic resources. The continuous increase in the number of hazelnut germplasm resources has made their preservation challenging. Additionally, some germplasm accessions suffer from synonymy or homonymy, with common mislabeling of germplasms, due to the widespread unlabeled propagation of grafting scions during the introduction of varieties across different regions, thereby hindering the identification, development, evaluation, and utilization of varieties.

The genetic diversity analysis based on DNA marker technology has become a powerful tool in the last two decades for identifying and evaluating germplasm resources. DNA fingerprinting has also emerged as an efficient technique for determining genetic diversity and distinguishing different varieties based on molecular markers or specific sequences [5]. Among these, simple sequence repeat (SSR) molecular markers are particularly useful for marker-assisted selection, especially in tree species with a long juvenile period such as hazelnuts [6]. Developing new Expressed Sequence Tag-Simple Sequence Repeat (EST-SSR) markers from RNA sequencing data of various hazelnut cultivars can increase the number of markers for fingerprinting and facilitate further genetic diversity analyses [7]. The Mehlenbacher lab at Oregon State University pioneered the development of SSR markers for hazelnuts. Subsequently, Bassil et al. [8] selected 53 polymorphic loci from the microsatellite-enriched libraries constructed by the lab, which included repeats of Glycine-Adenine (GAA), Cytosine-Adenine (CA), and Guanine-Adenine (GA). More than 200 SSR markers have been selected from these 3 libraries to date [9]. These SSR molecular markers are highly effective tools for assessing genetic relationships and parentage in *C*. *avellana* L. [10–12]. Furthermore, an analysis of the genetic diversity and structure in *C*. *heterophylla* and *C*. *kweichowensis* populations using these markers revealed the high genetic diversity of both populations. The clustering analysis distinctly segregated the 34 populations of *C*. *heterophylla* and *C*. *kweichowensis* into 2 major groupings [13]. Subsequent analysis of 12 *C*. *mandshurica* populations showed that these populations had high levels of genetic diversity. Further Mantel tests revealed a significant positive correlation between geographic and genetic distances among *C*. *mandshurica* populations [14]. Moreover, considering the simplicity of SSR band patterns, ease of statistical analysis, and interpretation, the International Union for the Protection of New Varieties of Plants recommends using SSR and SNP markers for constructing DNA fingerprinting profiles [15]. The fingerprinting capability of SSR markers can be determined based on their allele frequencies, making them more suitable for establishing varietal fingerprinting profiles. Recent studies have predominantly used SNPs to construct linkage maps for evaluating genetic diversity and investigating the domestication history of European hazelnuts [16]. SSR markers remain a valuable and cost-effective tool despite the significant cost reduction and advances in SNP genotyping. However, reports on using well-characterized SSR primer combinations for accession identification and fingerprinting profile construction in different hazelnut species are currently lacking. Therefore, the genetic diversity of hazelnut germplasm resources needs detailed exploration.

The concept of a core collection, initially introduced by Frankel and Brown, refers to a subset within a germplasm repository that maximally represents the genetic diversity of the repository while exhibiting minimal genetic redundancy [17]. Establishing a core collection for hazelnut resources can effectively reduce genetic redundancy and assist breeders in lowering management costs. Consequently, technical methods need to be employed to screen and construct a core set from the germplasm bank, thus ensuring the preservation of maximal genetic diversity. Core collections are typically constructed using two methodologies: phenotypic-based approaches and molecular marker–based genotypic data analyses. Phenotypic-based construction is susceptible to environmental influences, resulting in a higher error rate. Also, it necessitates the analysis of multiple traits, thereby imposing a substantial workload. DNA molecular marking technology is widely used in analyzing genetic diversity between different genera and in the construction of core germplasm owing to its high information content, efficiency, and independence from environmental factors. SSR markers possess high resolution and can accurately determine the size of target fragments, making them one of the most advantageous methods for constructing core collections [18–20]. SSR markers are now used to establish a core germplasm collection in European hazelnuts (*C*. *avellana* L.) [16, 21]. These accessions were selected as a core set to encompass the molecular genetics and morphological diversity in the national collection. This core set, characterized by its high genetic representativeness, has demonstrated significant benefits in resource acquisition, conservation, enhancement of breeding efficiency, and identification of critical traits.

Currently, reports on the systematic evaluation and core germplasm screening of hazelnut germplasm resources at the molecular level are scarce, significantly limiting the protection of hazelnut genetic diversity and the use of superior germplasm resources. Therefore, this study used fluorescent SSR markers for capillary fluorescent electrophoresis detection of hazelnut varieties in both the resource bank and external resources, aiming to analyze the genetic diversity and phylogenetic relationships of hazelnut varieties. Also, it selected highly polymorphic SSR primers to distinguish all 311 accessions with the fewest primers. Further, well-characterized SSR primer combinations were used to construct SSR fingerprinting profiles of hazelnut resources, establishing a reasonable primary core germplasm. This provides a scientific basis for breeding new superior varieties.

## Materials and methods

### Experimental materials and genomic DNA extraction

A total of 331 hazelnut resources were collected from the hazelnut national germplasm resource repository of the Liaoning Economic Forest Research Institute in Heishan County, the Songmu island research base, and Yichun City in Heilongjiang Province. These accessions included 163 cultivars and 146 landraces. Additionally, the 22 wild hazelnut accessions from Heilongjiang Province were used as an outgroup, which enhanced the phylogenetic accuracy of genetic analysis and ensured that the constructed core germplasm truly reflected the genetic diversity of the selected germplasm resources. The varieties under test primarily consisted of the Ping’ou hybrid hazelnut, a cultivated species of hazelnut with independent intellectual property rights in China, along with six different species from the genus *Corylus*, including *C*. *avellana*, *C*. *heterophylla*, *C*. *yunnanensis*, *C*. *sieboldiana*, *C*. *chinensis*, and *C*. *kweichowensis*, thereby exhibiting a rich genetic diversity. The material information is provided in S1 Table.

### Genetic diversity and primer polymorphism analysis

The genomic DNA was extracted from the test samples using an improved cetyltrimethylammonium bromide (CTAB) method, which omitted the selective precipitation and cesium chloride (CsCl) gradient steps, requiring only basic laboratory equipment. This modified approach enhanced efficiency and was well-suited for high-throughput extraction [22]. The quantity and purity of the extracted DNA were assessed using 1% agarose gel electrophoresis and spectrophotometry, respectively. The DNA concentration was adjusted to 50 ng/μL, and it was stored in an ultra-low-temperature freezer (Thermo Fisher Scientific, Karlsruhe Germany) at –80°C.

### SSR primer screening

A total of 33 pairs of SSR primers (S2 Table) developed from the genus *Corylus* were initially selected, referring to recently published relevant studies [1, 14, 23–25]. Also, 16 pairs of SSR primers with high polymorphism, strong stability, and good repeatability were identified through further screening (S3 Table). All primers were synthesized by Beijing Ruibio Biotech Co., Ltd. (Beijing, China).

### Fluorescent polymerase chain reaction amplification and capillary electrophoresis detection

Polymerase chain reaction (PCR) amplification was performed using DNA templates from 331 plants of the genus *Corylus*. The primer PCR amplification system was constructed using the M13 adapter method [26]. The total volume of the PCR reaction system for SSR primer screening was 20 μL, containing 30 ng of genomic DNA, 10 μL of 2×Taq PCR Mix (Takara Bio Inc.), 3.2 pmol of each reverse primer and fluorescently labeled M13 primer, and 0.8 pmol of forward primer with an M13 sequence at the 5’ end. The PCR reaction procedure was as follows: pre-denaturation at 95°C for 5 min, followed by 20 cycles of denaturation at 95°C for 30 s, annealing at 65°C for 30 s, and extension at 72°C for 30 s, and then 35 cycles of denaturation at 95°C for 30 s, annealing at 55°C for 30 s, and extension at 72°C for 30 s, concluding with a final extension at 72°C for 10 min. The 6-Carboxy-X-Rhodamine (ROX)-labeled PCR products were analyzed by capillary electrophoresis on an ABI 3730XL sequencer (Applied Biosystems, CA, USA).

### Data analysis

According to the format required by PowerMarker V3.25 software [27], the SSR original “bp type” data read from Excel spreadsheets was converted into PowerMarker format, with missing data assigned a value of 0. This software, along with GenAlEx6.5 [28], was used to calculate genetic diversity indices, including the number of alleles (*Na*), number of effective alleles (*Ne*), expected heterozygosity (*He*), observed heterozygosity (*Ho*), and Shannon’s information index (*I*). Also, the genetic distances between the varieties and the genetic similarity coefficient matrices derived from various primer combinations were analyzed. The Mantel correlation coefficient was used to assess the relationship between the genetic similarity matrix of each primer pair and the overall similarity matrix across all primers. Further, the clustering results were obtained using MEGA 7.0.26 [29] to create visual dendrograms and the unweighted pair group method with arithmetic mean (UPGMA), saved in Newick format, and uploaded to the Interactive Tree of Life website www.itol.com for beautification. Primer combinations with distinct amplification peaks and high polymorphism were selected based on these results, and the product fragment sizes from each individual under different primer amplifications were recorded to create fingerprint profiles.

### Construction of primary core collection

The optimized 11 pairs of core primers were used to screen the primary core collection to ensure the representativeness, economy, and efficiency maximization of SSR primer combinations. These were analyzed using the PowerCore v.1.0 software based on allele maximization (M strategy) and a heuristic algorithm [30]. While constructing the core collection, the software verified the distribution of marker allele frequencies to ensure dataset reliability. It automatically generated appropriate sampling ratios, thus eliminating the need for manual input. Subsequently, the core collection was generated and assessed for genetic diversity using Nei and Shannon–Weaver diversity indices. The genetic diversity was used to determine the primary core and original collections. A *t* test was used to detect significant differences in genetic diversity information between the two collections. Additionally, principal coordinate analysis (PCoA) was performed using GenAlEx 6.503 software to confirm the successful construction of the primary core collection [31].

## Results

### Genetic diversity and primer polymorphism

**Fig 1:**
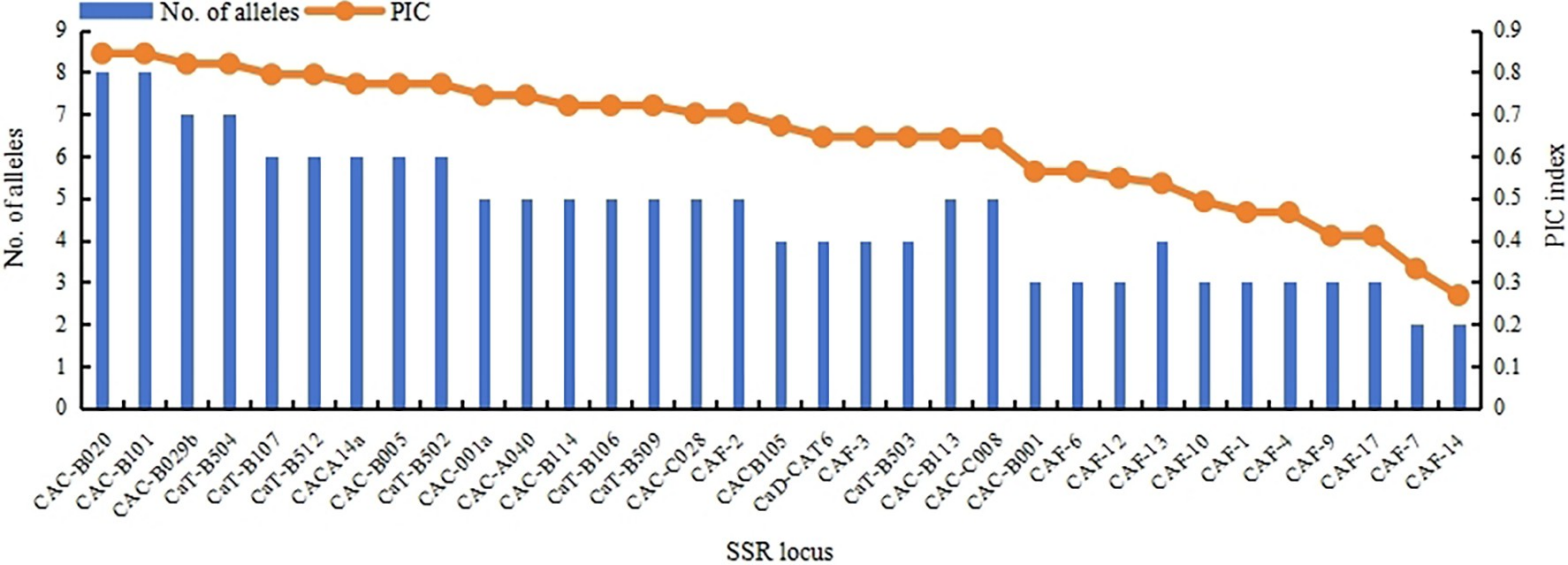
Variation in allele numbers and PIC indices for 33 SSR primers.

The preliminary results from fluorescent capillary electrophoresis revealed significant differences in polymorphic information content (PIC) values and the number of alleles among different primers in the samples. No positive correlation was observed between these values, which was related to the genetic background of the amplified samples. Primers with the same PIC value indicated that the resolution efficiency of the two sets of markers, such as CAC-A14a and CAC-B005, was consistent. Fig 1 shows the amplification effects of 33 pairs of test primers on different samples. Subsequently, 16 primer pairs with good reproducibility and high specificity were selected from 33 pairs, prioritizing those with the highest PIC values for genetic diversity analysis. As delineated in Table 1, these 16 primer pairs collectively identified 232 *Na* across the samples, averaging 14.5 alleles per locus. The *Ne* value ranged from 3.154 to 7.130, with a mean value of 5.313. The average observed values for *Ho*, *He*, and *I* were 0.746, 0.797, and 1.903, respectively, indicating a high level of genetic diversity among current hazelnut species. The PIC value ranged from 0.634 to 0.851, with all primer PIC values greater than 0.5. It indicated that all primers selected in this study had high polymorphism and were suitable for the identification and genetic diversity analysis of 331 hazelnut materials. The average values for *Ho*, *He*, and *I* were 0.746, 0.797, and 1.903, respectively, indicating a high level of genetic diversity among current hazelnut species. The PIC value ranged from 0.634 to 0.851, with all primer PIC values greater than 0.5, indicating that all primers selected in this study had high polymorphism and were suitable for the identification and genetic diversity analysis of 331 hazelnut materials.

**Table 1:** Polymorphism analysis of 16 SSR primers.

Marker	*Na*	*Ne*	*I*	*Ho*	*He*	MAF	Heterozygosity	PIC
CAC-001a	11	3.505	1.601	0.775	0.715	0.449	0.761	0.693
CAC-A040	10	3.243	1.398	0.683	0.692	0.461	0.677	0.654
CACA14a	14	5.34	1.92	0.685	0.813	0.313	0.677	0.796
CAC-B005	13	5.825	1.973	0.872	0.828	0.251	0.867	0.809
CAC-B020	25	6.27	2.21	0.79	0.841	0.302	0.782	0.828
CAC-B029b	17	6.477	2.208	0.942	0.846	0.284	0.94	0.831
CAC-B101	17	6.282	2.174	0.802	0.841	0.27	0.798	0.826
CAC-B114	9	3.154	1.33	0.299	0.683	0.423	0.296	0.634
CAC-C028	10	3.733	1.526	0.727	0.732	0.381	0.725	0.693
CAF-2	14	4.185	1.739	0.485	0.761	0.375	0.474	0.742
CaT-B106	15	6.827	2.101	0.948	0.854	0.201	0.927	0.844
CaT-B107	16	7.13	2.221	0.892	0.86	0.221	0.876	0.851
CaT-B502	16	6.604	2.19	0.865	0.849	0.284	0.834	0.845
CaT-B504	15	6.415	2.121	0.93	0.844	0.284	0.927	0.829
CaT-B509	11	5.011	1.792	0.842	0.8	0.287	0.837	0.776
CaT-B512	19	5.008	1.939	0.393	0.8	0.264	0.39	0.777
Total	232	85.009	30.44	11.931	12.757	5.048	11.789	12.426
Mean	14.5	5.313	1.903	0.746	0.797	0.316	0.737	0.777

*He*, expected heterozygosity; *Ho*, Observed heterozygosity; *I*, Shannon’s diversity index; *Na*, number of alleles; *Ne*, effective number of alleles; PIC, polymorphic information content.

### Clustering of genetic relationships

**Fig 2:**
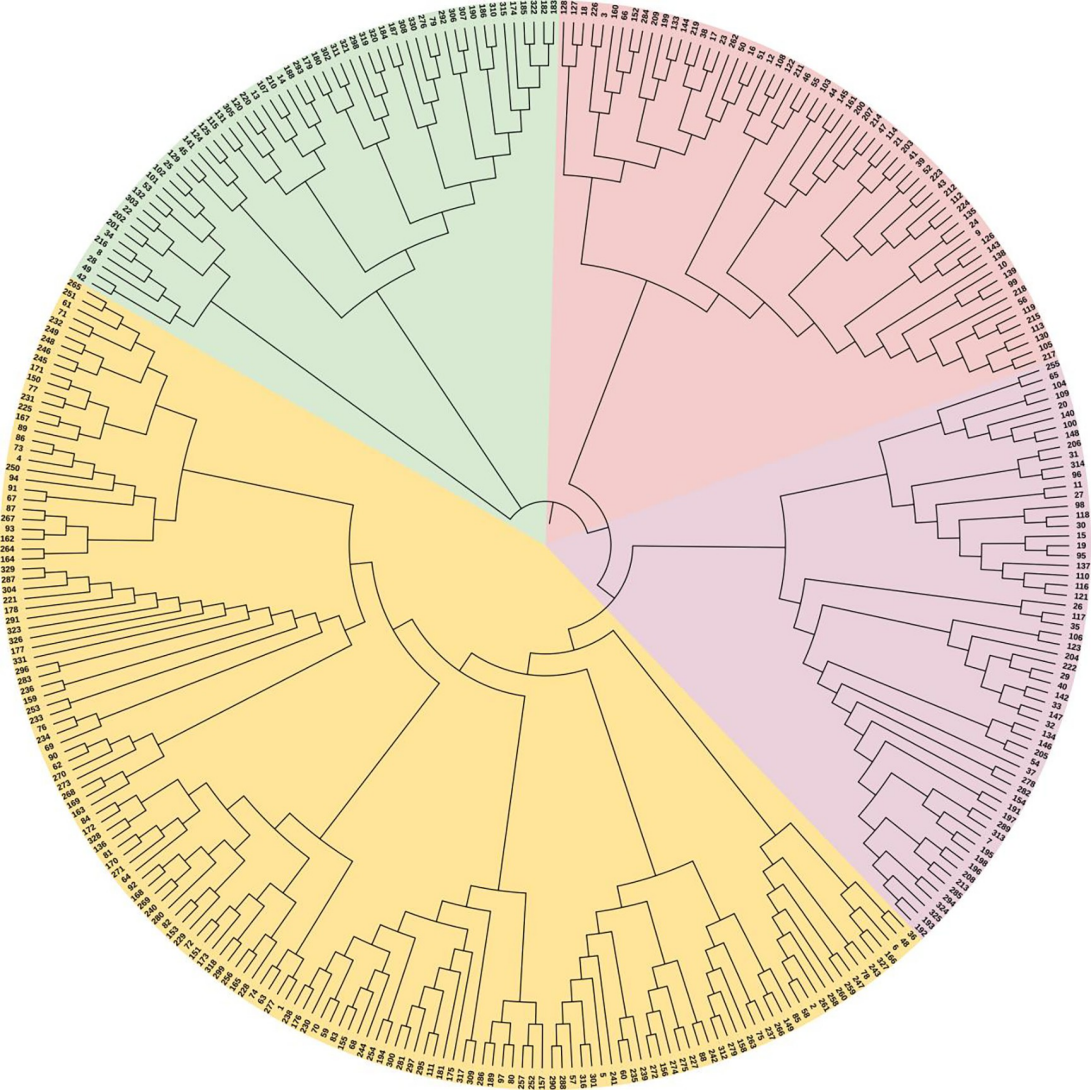
Phylogenetic relationship of 331 *Corylus* species, with four different colors indicating four groups.

A diversity analysis of the tested resources revealed that the selected primers exhibited high polymorphism in 331 individuals. A phylogenetic tree was constructed using the UPGMA method based on Nei’s genetic distance to further explore the genetic relationships among 309 germplasm resources and 22 *Corylus* L. accessions from Heilongjiang Province as an outgroup (Fig 2). The branch lengths in the figure represent the genetic variation and distance between materials. Shorter branches indicate closer genetic distances, lower genetic variation, and closer genetic relationships. All materials were categorized into four distinct clusters based on the outcomes of the cluster analysis. The primary cluster, designated in purple, predominantly comprised 47 Ping’ou hybrid hazelnuts and 10 germplasm resources originating from outside the province. The clustering outcomes suggested that most hybrid varieties exhibited close genetic relationships and elevated genetic similarity. The second cluster, marked in red, encompassed 63 Ping’ou hybrid hazelnut materials, primarily derived from the crosses conducted between 1981 and 1985. However, the grouping analysis did not reveal a consistent correlation between the clustering results and the years of hybridization. The third major cluster (purple) and the fourth cluster (yellow) encompassed 61 and 150 *Corylus* species, respectively. It predominantly comprised samples derived from crosses between *C*. *heterophylla* and *C*. *avellana*, and their interspecific hybrids. Among the samples, the Ping’ou hybrid hazelnut varieties Xianda No. 1 and 85–8 demonstrated the closest phylogenetic relationship, with a genetic distance of 0.5078. Cluster IV was further subdivided into six subgroups, containing 14, 26, 27, 28, 26, and 29 materials, respectively, each exhibiting more pronounced genetic affinities within the subgroup. Furthermore, a cross-distribution of *C*. *heterophylla*, *C*. *avellana*, and hybrid hazelnuts was observed across different subgroups, which might be attributed to the limited number of primer sets used for identification, as well as the ongoing exchange and hybridization among varieties. In cluster IV, subgroup 6, the genetic distances among the cultivars Barcelona, OSU 104E, OSU BS, and OSU 18 were extremely low, suggesting that these four cultivars likely originated from a common ancestor. The clustering results objectively reflected the phylogenetic relationships among the cultivars. Integrating SSR genetic diversity data with morphological diversity assessments can provide more comprehensive and in-depth information on germplasm resources, thereby facilitating the breeding of superior cultivars and the sustainable management of genetic resources in the future.

### Core primer screening

A Mantel’s test was conducted to assess the correlation between the genetic similarity coefficient matrices derived from 16 pairs of primer combinations and the parent matrix. This analysis aimed to optimize the selection of genetic markers, thereby enhancing the precision and efficiency of marker screening. The results of the correlation analysis are detailed in Table 2. The primer count was progressively decreased according to the ascending order of correlation within the genetic similarity coefficient matrices. The study revealed no change in the number of identified varieties and no significant differences in the genetic similarity coefficients between varieties when the primer count was trimmed to 11 pairs. Also, the clustering results remained coherent, effectively representing genetic diversity among varieties. However, reducing the primer count to 10 pairs or fewer resulted in fewer identified varieties and chaotic clustering results. Given the discrimination rate, PIC value, Shannon’s index, and stability of these 11 pairs of highly polymorphic primers for identifying hazelnut accessions, it is deemed feasible to employ these 11 pairs as core primers for accession identification and suitable for variety identification and genetic differentiation.

**Table 2:** Correlation analysis of genetic correlation coefficients.

Primer name	CAC-001a	CAC-A040	CAC-A14a	CAC-B005	CAC-B020	CAC-B029b	CAC-B101	CAC-B114	CAC-C028	CAF-2	CaT-B106	CaT-B107	CaT-B502	CaT-B504	CaT-B509	CaT-B512
Correlation coefficient	0.482**	0.373**	0.389**	0.441**	0.431**	0.370**	0.478**	0.306**	0.438**	0.267**	0.437**	0.421**	0.321**	0.501**	0.415**	0.454**

Correlation coefficient represents the strength of correlation between genetic markers. The closer the correlation coefficient is to zero, the weaker the correlation; ** means the correlation was extremely significant at *P* <0.01 level.

### Construction of SSR fingerprinting

A total of 11 SSR molecular markers were obtained through primer screening, which were then used to detect loci in hazelnut varieties, resulting in 164 alleles composed of polymorphic and reproducible allelic sites. The matching analysis of varieties was performed in GenAlEx6.51 based on allelic locus data. Also, no two varieties with completely identical loci were detected, indicating that each material had a unique combination of SSR multi-locus genotypes. This allowed for the complete differentiation of all 331 hazelnut resources. An SSR fingerprint map (S1 Fig) was constructed using this set of primer combinations, with the horizontal axis representing marker numbers, the vertical axis representing variety numbers, and the legend indicating the size of the amplified fragments. The resources were distinguished, and precise identification was achieved using this SSR fingerprint map.

### Screening of primary core germplasm resources

PowerCore was used to calculate the core subset and the number of original varieties based on the genetic distance matrix between resources. As shown in Table 3, the Shannon–Weaver and Nei indices of the core germplasm were generally higher than those of the original germplasm, indicating that the core germplasm exhibited a higher level of genetic diversity. The original germplasm comprised 331 varieties, and the primary core germplasm constructed at a sampling ratio of 38.36% included 127 germplasm varieties (Table 4). At this point, the number of allelic sites in the primary core germplasm was the same as in the original germplasm. Thus, the core resources obtained through screening encompassed the genetic diversity of all resources, representing the allele number of all resources. Additionally, the efficiency index was 0.86 during the screening process, indicating that 86% of the core germplasm resources were effectively retained, thus implying that the core germplasm could adequately represent the genetic diversity of the original resources with a smaller sample size.

**Table 3:** Statistical analysis of genetic diversity parameters in core and original germplasm collections.

Character	C-Sh.W.	C-Nei	C-Allele	E-Sh.W.	E-Nei	E-Allele	Character	C-Sh.W.	C-Nei	C-Allele	E-Sh.W.
CaT-B509	3.173	0.945	35	2.88	0.919	35	CaT-B509	3.173	0.945	35	2.88
CaT-B107	3.818	0.973	58	3.429	0.945	58	CaT-B107	3.818	0.973	58	3.429
CaT-B502	3.868	0.973	63	3.541	0.956	63	CaT-B502	3.868	0.973	63	3.541
CAC-001a	2.904	0.909	33	2.498	0.846	33	CAC-001a	2.904	0.909	33	2.498
CAC-C028	2.818	0.923	25	2.473	0.878	25	CAC-C028	2.818	0.923	25	2.473
CACA14a	3.178	0.927	41	3.073	0.931	41	CACA14a	3.178	0.927	41	3.073

For each locus: C-Sh.W., Shannon’s diversity index for the core collection; C-Nei, Nei’s gene diversity index for the core collection; C-Allele, number of alleles in the core collection; E-Sh.W., Shannon’s diversity index for the original germplasm collection; E-Nei, Nei’s gene diversity index for the original germplasm collection; E-Allele, number of alleles in the original germplasm collection.

**Table 4:** Comparison of genetic diversity among *Corylus* core and original collections.

Population	Number	Percentage (%)	Heterozygosity	*PIC*	*Ne*	*I*	*Ho*	*He*	MAF
Original collection	331	100	0.7034 ± 0.17a	0.757 ± 0.07a	4.951 ± 1.46b	1.827 ± 0.33a	0.713 ± 0.18a	0.781 ± 0.06a	0.342 ± 0.08a
Core collection	127	38.36	0.7058 ± 0.20a	0.728 ± 0.08a	6.204 ± 2.66a	2.003 ± 0.41a	0.711 ± 0.16a	0.806 ± 0.08a	0.361 ± 0.07a

The data are expressed as mean ± standard deviation (*n* = 331). Different letters indicate statistically significant differences (*P* < 0.05).

A comparison of the genetic diversity parameters between the constructed core and original subsets showed that the PIC (0.728) and *Ho* (0.711) values of the core germplasm were smaller than those of the original germplasm (0.757 and 0.713). In contrast, the remaining five genetic parameters were greater than those of the original germplasm. A *t* test on the seven genetic parameters revealed that only *Ne* was significantly different (*P* < 0.05) between the primary core and original germplasms. These tests indicated that the primary core germplasm constructed based on a compression ratio of 38.36% could adequately represent the genetic diversity of the original germplasm and was confirmed as the final core germplasm constructed in this study.

**Fig 3:**
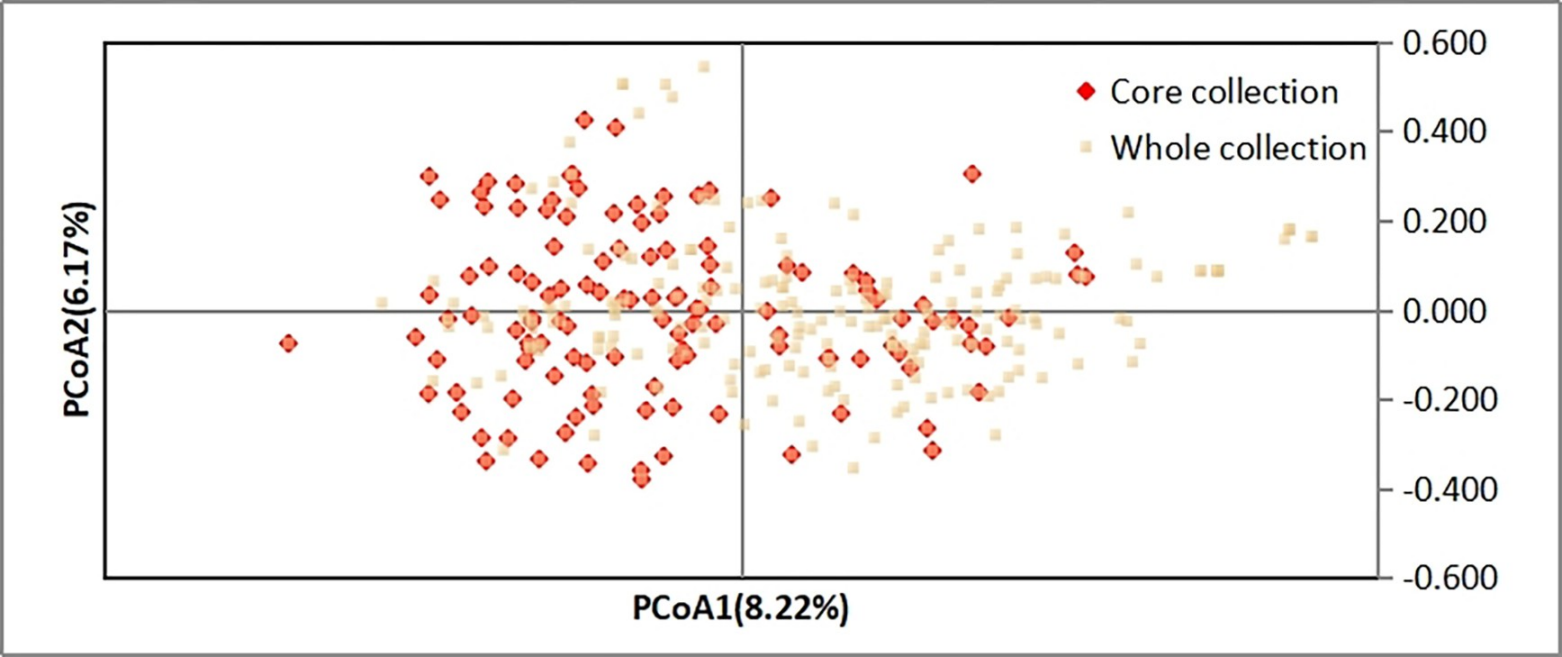
Analysis of principal coordinate plots of core and original collections. Red represents the variety of core collection, and white represents the whole collection.

The genetic diversity of the core and original collections was assessed using PCoA to determine the even distribution of the core collection (Fig 3). The principal component analysis of germplasm resources showed that the first and second principal components explained 6.17% and 8.22% of the total variation, respectively. The positional distance on the two-dimensional plane indicated the degree of relatedness between germplasms. The PCoA analysis revealed that the constructed core germplasm not only resembled the distribution of the original germplasm but also exhibited a more uniform and comprehensive distribution compared with the entire resource.

A total of 67 cultivars, 43 landraces, and 17 accessions were included in the core collection. S4 Table presents detailed information regarding the core germplasm. These 127 core germplasms of hazelnut effectively represented the genetic diversity of the original germplasm. These core germplasms can be effectively used to aid in discovering new genes and cultivating new varieties by establishing a core germplasm to eliminate genetic redundancy and duplication in the original germplasm resources and subsequently increase the retention rate of genetic diversity parameters.

## Discussion

### Assessing the genetic diversity of *Corylus* L. populations

The rich and diverse forest tree germplasm resources serve as the raw material for tree genetic improvement and elite variety selection, also forming the basis for genetic and species diversity. The hazel species from abroad was introduced into China at the end of the 19th century. However, these introduced resources have not developed on a significant production scale over time. These varieties have been officially approved, and a national “legal pass” has been granted for their promotion and cultivation. The substantial germplasm resources collected in this study included 260 individual samples of *C*. *heterophylla* Fisch. × *C*. *avellana* L. hybrids, sourced from the hazelnut national germplasm resource repository, which exceeded the number collected for other hazel species. Despite this, the information regarding the genetic diversity of hazel germplasm resources and the extent of their high-quality resource utilization is still quite limited. SSR marker technology has been widely applied in the fields of genetic diversity and identification of the genus *Corylus* in recent years due to its co-dominance, high polymorphism, and reproducibility [25, 32]. This study used 16 pairs of primers derived from European hazelnut and Ping’ou hybrid hazelnuts to analyze the genetic diversity and variety identification of 331 hazelnut materials. The average *Na* and *Ne* values were 14.5 and 5.313, respectively, indicating a high level of genetic diversity in the selected varieties, which was similar to the findings of Zong [14]. This might be attributed to the choice of materials for this study, as *Corylus* spp. are typically wind pollinated, which theoretically enhances their genetic diversity. Furthermore, the genetic materials selected are widely distributed, encompassing strains from various regions. These hazelnut materials possess a large number of alleles and a high level of genetic diversity as a result of early-stage comprehensive polymorphism screening of SSR primers.

### Screening of core primers and variety identification

Genetic relationships are influenced by two primary factors in the molecular identification of varieties: SSR primers and the number of samples in the identification group. Ma Qinghua et al. used 12 pairs of developed SSR primers to identify domestic Ping’ou hybrid hazelnut varieties. The results indicated that primer combinations with high PIC values could completely distinguish 43 Ping’ou hybrid hazelnut varieties, with the combination of primers CAF-2 and CAF-13 effectively identifying currently promoted varieties [25]. Eleven representative core primers were optimized according to genetic coefficients based on 16 pairs of primers in this experiment, which could distinguish all species. This method reduced the number of primers, thereby decreasing experimental costs and operational complexity. However, none of the primers could distinguish between varieties 84–22 and 85–127, indirectly proving their common origin. Concurrently, increasing the number of primers for analysis is necessary when identifying numerous varieties, especially when the samples are closely related. Also, considering specific primers with lower PIC values is essential to determine whether a phenomenon of synonyms existed [33]. The representativeness of primers is crucial while examining genetic variation relationships. Effective reflection of genetic relationships between varieties requires several variable primer sites (S5 Table). Additionally, the allele site analysis between different varieties reveals that each group possesses unique allele sites and frequency differences of shared sites, which are potentially attributable to distinct geographical, environmental conditions and seedling selection processes [19]. The subsequent step involves screening for core primers with strong representativeness, good specificity, and uniform distribution to construct different types of core primer databases tailored to the specific needs and content of various research groups. Furthermore, this study used 11 core primers to construct a visualized DNA fingerprinting profile for hazelnut cultivars, distinguishing all varieties. This fingerprinting profile provided robust support for identifying, conserving, and utilizing hazelnut germplasm resources.

### Construction of core germplasm

Core germplasm resources serve as representative subsets of the entire germplasm, preserving the maximum genetic potential while eliminating redundant resources. Hazelnuts, as monoecious, cross-pollinated plants, typically exhibit complex genetic relationships while selecting superior materials. Consequently, hazelnut germplasm materials from different varieties or geographical origins often cluster distinctly during cluster analysis. A strategy based on CoreFinder’s maximization (M strategy), inspired by the nonpopulation approach used in constructing the Huangqi (*Astragalus* spp.) core collection, has been implemented to optimize the collection and management of hazelnut germplasm [34]. This strategy preserves all alleles and typically yields a core collection with higher genetic diversity parameters than the original collection. The genetic bottlenecks arising from genetic relationships can be effectively mitigated by employing the M strategy, ensuring a broad and rich genetic foundation for the selected breeding materials.

In this study, an efficient germplasm selection strategy was employed to optimize the utilization and management of the hazelnut germplasm repository. This repository is comprised of a diverse array of cultivars and varieties, along with an additional 22 germplasm resources from Heilongjiang Province that serve as outgroup resources. A core collection preserving 100% of the allelic loci was successfully established using only 38.36% of the original germplasm, revealing that the genetic diversity of the core collection was representative of the entire collection. This result was consistent with known core germplasm construction ratios, where the core germplasm typically accounted for 5%–40% of the original germplasm in various plants, with exceptional core germplasm ratios potentially reducing to 5%–15% [35].

It is crucial to recognize that the genetic diversity of germplasm resources cannot be solely represented by agronomic and morphological data; SSR molecular markers are effective in representing this diversity. Based on this, a significant number of agronomic and morphological traits of genus *Corylus* can be preserved using the 127 primary core germplasm as a foundation, providing a reasonable reference for identifying, managing, and using hazelnut germplasm. Furthermore, with the continuous evolution of market demands and breeding efforts, the emergence of more hazelnut cultivars with superior traits and rich genetic variation is anticipated in the future. Consequently, ongoing research will necessitate the continuous refinement of the core germplasm.

## Conclusions

This study involved a comprehensive genetic diversity assessment on 331 hazelnut germplasms, including cultivars and landraces, using highly polymorphic primers to establish an SSR fingerprinting database and a core collection for hazelnut germplasm resources. The results provided essential data for conserving and exploiting hazelnut genetic diversity, offering significant insights for germplasm preservation, genetic studies, and breeding initiatives.

## Supporting information

S1 Table Essential information of 331 varieties. (XLSX)

S2 Table Essential information of 34 primers. (XLSX)

S3 Table Information of the SSR markers for analysis. (XLSX)

S4 Table Detailed information of core germplasm. (XLSX)

S5 Table Allele sizes of 331 hazelnut accessions at 16 SSR loci. (XLSX)

S1 Fig Fingerprinting of 331 hazelnut cultivars. (DOCX)
