# Supplementary material for: Utilizing SSR-based core collection development to improve conservation and utilization of Corylus L. genetic resources
Source: PLoS One. 2024 Oct 29;19(10):e0312116. doi: 10.1371/journal.pone.0312116 (PMC11521296; doi:10.1371/journal.pone.0312116)
Supplement: S1 Fig — (DOCX) [file pone.0312116.s006.docx]

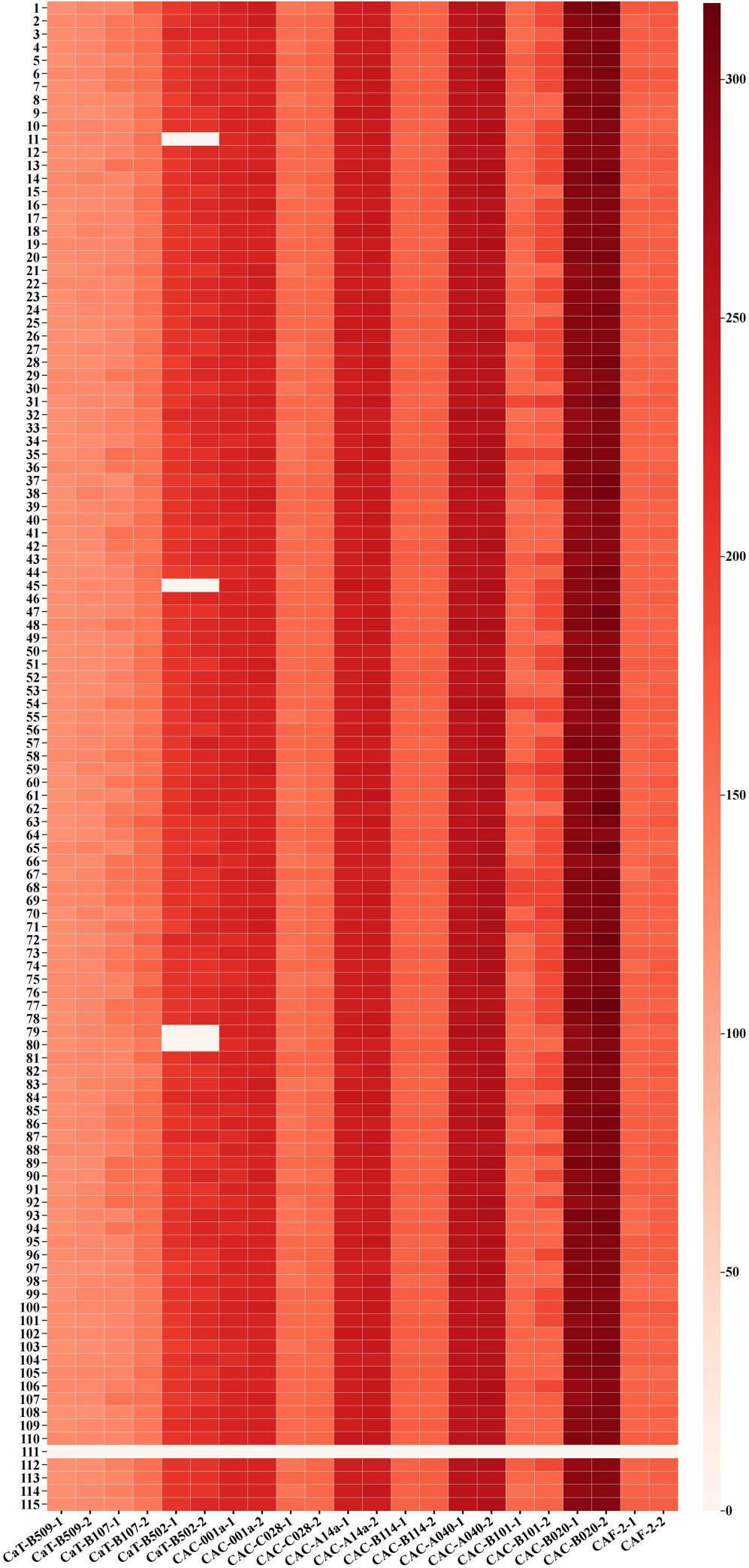

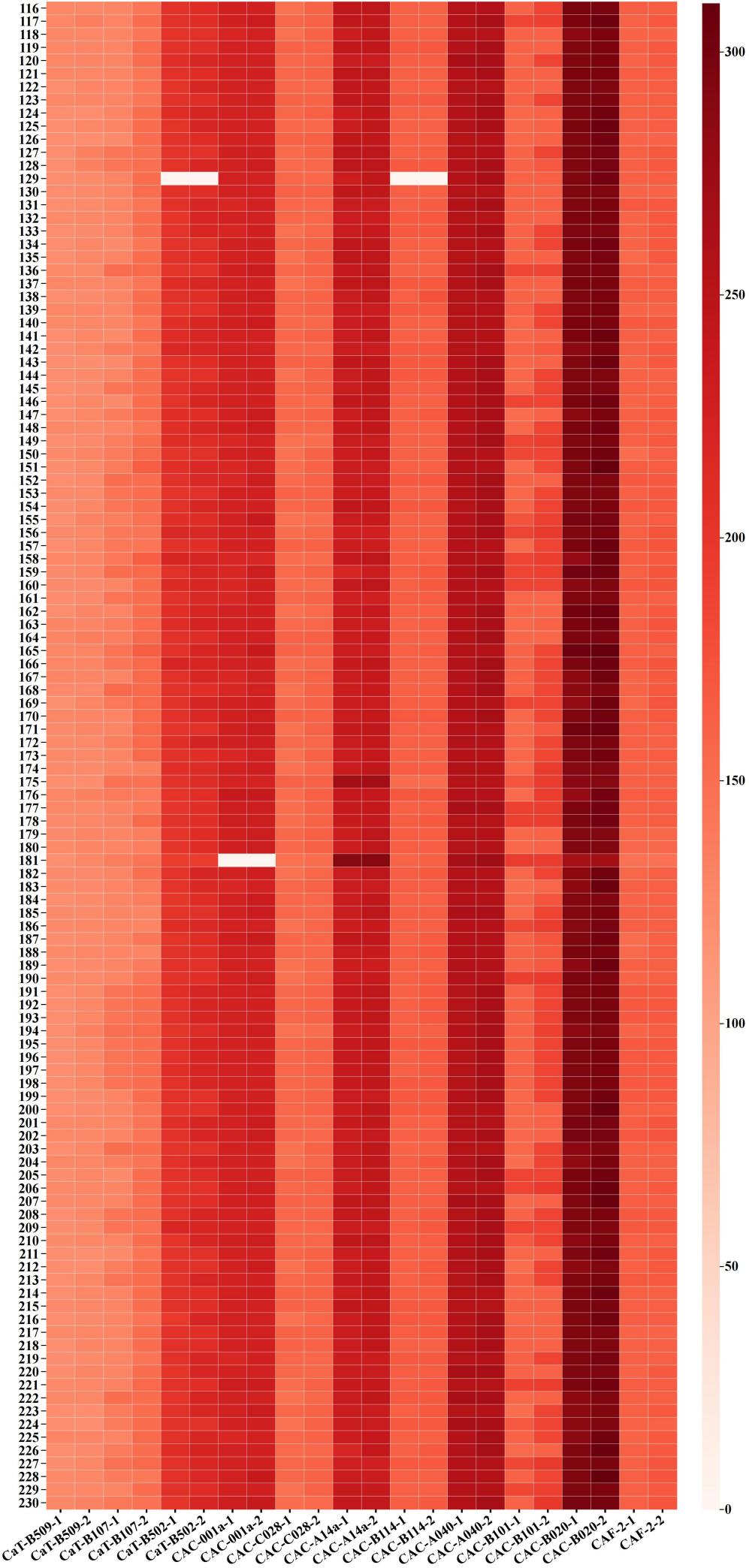

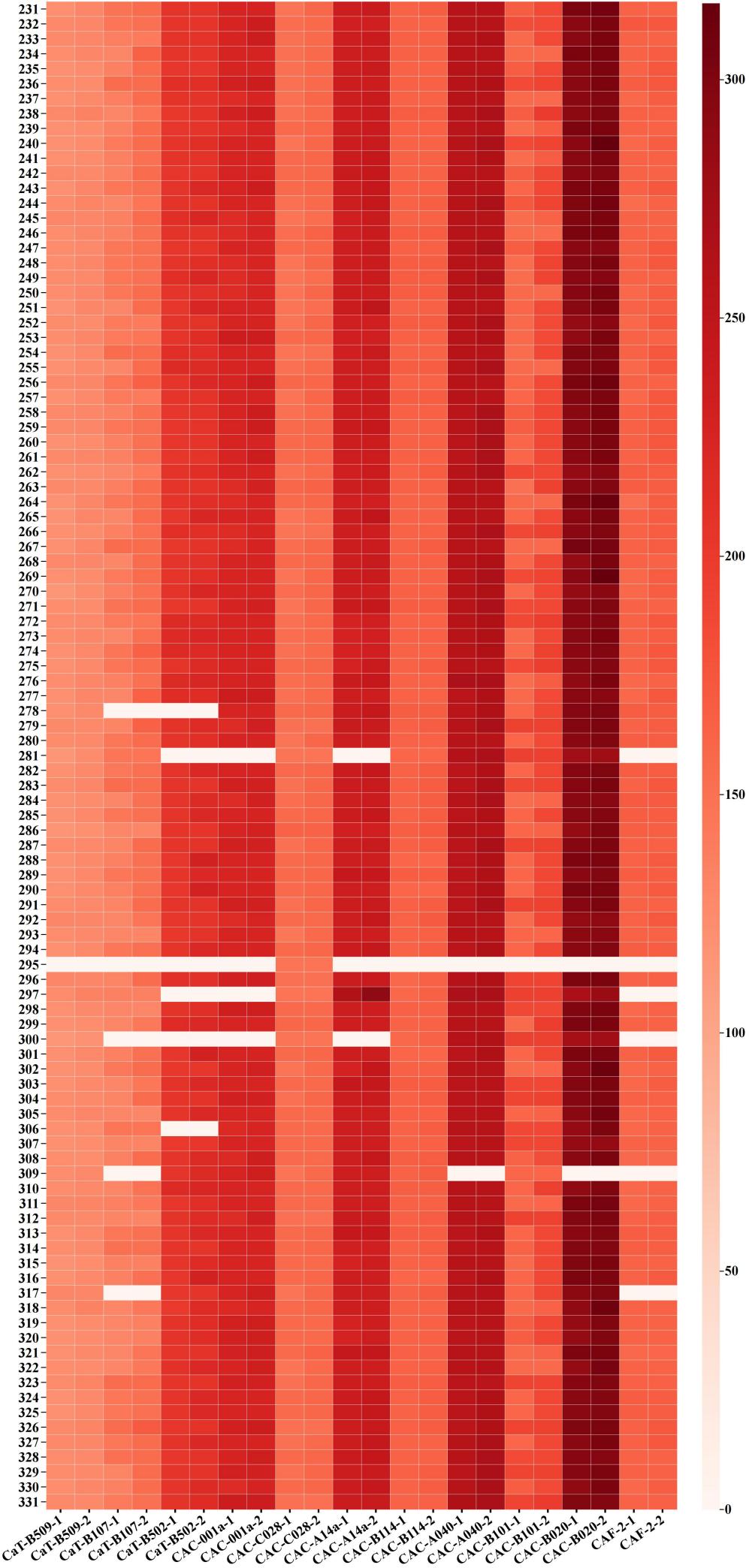


A

B

C

**S1 Figure. Fingerprinting of 331 hazelnut cultivars.**

Each colored box within panels (A), (B), and (C) denotes an allele, with white boxes signifying deletion loci. The vertical axis corresponds to the variety identifiers, whereas the horizontal axis indicates the core marker indices.
